# Naphthenic Acid Corrosion Mitigation: The Role of Niobium in Low-Carbon Steel

**DOI:** 10.3390/ma17133372

**Published:** 2024-07-08

**Authors:** Nurliyana Mohamad Arifin, Kesahvanveraragu Saravanan, Ervina Efzan Mhd Noor

**Affiliations:** 1 Centre for Manufacturing and Environmental Sustainability (CMES), Multimedia University, Bukit Beruang 75450, Malaysia; 2 Faculty of Engineering and Technology, Multimedia University, Bukit Beruang 75450, Malaysia; kesahvanveraragu@gmail.com

**Keywords:** pipeline steel, corrosion, low-carbon steel, reinforcement of niobium, corrosion rate, microstructural, chemical composition, naphthenic acid

## Abstract

Naphthenic acid corrosion is a well-recognized factor contributing to corrosion in the construction of offshore industry pipelines. To mitigate the corrosive effects, minor quantities of alloying elements are introduced into the steel. This research specifically explores the corrosion effects arising from immersing low-carbon steel, specifically A333 Grade 6, in a naphthenic acid solution. Various weight percentages of niobium were incorporated, and the resulting properties were observed. It was noted that the addition of 2% niobium in low-carbon steel exhibited the least mass loss and a lower corrosion rate after a 12 h immersion in naphthenic acid. Microstructural analysis using scanning electron microscopy (SEM) revealed small white particles, indicating the presence of oil sediment residue, along with corrosion pits. Following the addition of 2% niobium, the occurrence of corrosion pits markedly decreased, and only minor voids were observed. Additionally, the chemical composition analysis using energy-dispersive X-Ray analysis (EDX) showed that the black spot exhibited the highest percentage of carbon, resembling high corrosion attack. Meanwhile, the whitish regions with low carbon content indicated the lowest corrosion attack. The results demonstrated that the addition of 2% niobium yielded optimal properties for justifying corrosion effects. Therefore, low-carbon steel with a 2% niobium addition can be regarded as a superior corrosion-resistant material for offshore platform pipeline applications.

## 1. Introduction

Corrosion refers to the detrimental and undesired impact of a corrosive atmosphere on metals and alloys, leading to a reduction in the lifespan of materials utilized in the fabrication of offshore pipelines [1]. Due to the deterioration of metal surfaces, materials may experience a decline in their mechanical, as well as other physical, chemical, and physicochemical properties, along with alterations in their appearance [2]. The substantial economic impact of corrosion extends its influence across various industries, domestic uses, and public sectors globally, underscoring the imperative for enhanced corrosion control measures. Efficient corrosion inhibition holds significant economic value, given that the estimated annual cost of corrosion is anticipated to reach 3–4% of the gross domestic product (GDP) in developed nations. Within the realms of the oil, gas, and chemical industries, corrosion stands out as one of the most formidable challenges, with an estimated annual cost of 170 billion USD. The impetus for advancing corrosion-resistant materials and enhancing global corrosion mitigation strategies is not solely driven by the substantial financial burden imposed by corrosion [3]. The health and environmental risks linked to the potential failure of oil and gas equipment further propel these developments on a worldwide scale.

Crude oil is a complex mixture of three major hydrocarbons, which are paraffins, naphthene, and aromatics compounds. The hydrocarbon content in crude can range from as high as 97% in lighter paraffinic crude to as low as 50% in heavier and bitumen crude. Naphthenic acids, which are organic acids found in naphthene compounds, are present in crude oil and certain refined petroleum products [4]. Naphthenic acid corrosion, also known as NAC, is a form of corrosion that occurs in industrial equipment, particularly in oil refining and processing facilities, due to the presence of naphthenic acids [5]. When these acids come into contact with steel or other metallic materials in equipment such as pipelines, tanks, and processing units, they can cause corrosion. The quality of the crude oil is the greater part of the reason for corrosion problems, where the presence of tar allows for confirmation of the presence of naphthenic acid with high percentages [6]. Naphthenic acid is frequently present in crude oil throughout the refining process, leading to simultaneous occurrences of naphthenic acid corrosion that interact within the entire crude oil distillation units. This issue has not been fully explored, but its significant impact on the production operations of oil refinery enterprises is evident [7]. Indeed, the temperature plays a crucial role during the flow, not only enhancing the fluidity of the oil and accelerating chemical reactions but also activating hazardous impurities. Thus, these reactions contribute to the promotion of corrosion [8].

The predominant utilization of carbon steel in oil field pipelines is primarily driven by economic and strength considerations. Consequently, the corrosion rate of carbon steel is not solely determined by electrolyte conditions but can also be affected by its chemical composition and microstructure [9]. Carbon steel is a material primarily composed of carbon as its principal alloying component, comprising iron (Fe), carbon (C), manganese (Mn), phosphorus (P), sulfur (S), and silicon (Si) [10]. Low-carbon steel, commonly referred to as mild steel, typically comprises less than 0.3% carbon. In contrast, medium- and high-carbon steels have carbon contents ranging between 0.3 and 0.45%, and 0.45 and 0.75%, respectively. In the pipeline industry, particularly for offshore pipelines, the avoidance of medium- and high-carbon steel is attributed to their diminished resistance to brittleness and compromised weldability [11]. Low-carbon steel type ASTM A333 Grade 6 is a standard specification for seamless and welded steel pipe for low-temperature service [12]. The chemical composition and mechanical characteristics for this steel is shown in Table 1.

Compared to other metallic or non-metallic materials, carbon steels have a low corrosion resistance. According to the literature, various methods have been attempted to improve the corrosion resistance of carbon steel. However, most of them are limited by the high manufacturing costs or complexity of the technologies [13]. Nevertheless, one method that showed promising results was the introduction of minor quantities of alloying elements. Several elements, such as chromium (Cr) [14], nickel (Ni) [15], copper (Cu) [16], molybdenum (Mo) [17], tin (Sn) [18], and niobium (Nb) [19], are added to low-carbon steel as microalloying agents in order to obtain enhanced mechanical or corrosion resistance properties. Niobium (Nb) microalloying is a promising method to control the recrystallization process in steels. Niobium is classified as a rare metal owing to its remarkable characteristics, such as a high melting point, corrosion resistance, and its ability to form a range of crucial alloys for industrial use. The addition of Nb in carbon steel contributes to strong solute drag and nitrite–carbonate precipitates pinning and generates an almost pure ferrite matrix in Nb-bearing steel by grain refinement and dispersion strengthening [20]. According to Torkamani et al., it was noted that presence of rare earth elements, such as Nb, has an impact on the microstructure and impact toughness of cast ferritic–pearlitic micro-alloyed steels. The alteration in inclusions and their influence on the solidification process, including peritectic transformation, affects the impact toughness of the cast steel [21]. Furthermore, Nguyen et al. found that potentiodynamic testing demonstrates passive behavior and a decline in passive currents as the Nb content increases. These findings imply that the interaction of Nb enhances the corrosion resistance of low-alloy steel [22]. Furthermore, findings from Gao et al. indicate that the addition of Nb is successful in refining the grains and microstructure, leading to the development of fine precipitates in low-carbon micro-alloyed cast steels. However, when the Nb content surpasses 0.044 wt.%, its efficacy in grain refinement diminishes. This is attributed to the fact that the amount of dissolved Nb does not increase further, and Nb-containing second-phase particles may undergo coarsening [23]. Therefore, niobium is considered advantageous alloying element, contributing to the improvement in steel strength while simultaneously enhancing its corrosion resistance.

This study focused on examining the corrosion effects by immersing low-carbon steel (Fe-C), specifically A333 Grade 6, in a naphthenic acid solution. Different weight percentages of niobium (Nb) were incorporated into the low-carbon steel, yielding a modified composition referred to as Fe-C-Nb. The evaluation of corrosion impact, measured in terms of mass loss and corrosion rate, was conducted. Furthermore, the microstructure and elemental composition of Fe-C-Nb underwent detailed characterization.

## 2. Method and Materials

The selection of low-carbon steel type A333 Grade 6 (Liaocheng Huililai Import and Export Co., Ltd., Liaocheng, China) for this study was motivated by its appropriateness for low-temperature applications, as it maintains impact toughness even at temperatures as low as −45 °C. Furthermore, niobium metal powder was chosen to enhance the low-carbon steel. The fabrication of Fe-C-Nb encompassed multiple stages, involving the preparation of low-carbon steel samples, niobium powder preparation, the melting procedure, and lost-foam casting. Initially, A333 Grade 6 steel was measured and cut into four segments, each with a length of 8 cm, to facilitate the melting process. These steel samples were weighed, and the measurements were meticulously recorded. Subsequently, three distinct amounts of niobium metal powder were accurately weighed and stored in sealed bags. The calculation of the niobium metal powder weight was determined in accordance with Equation (1) [24].

(1) $$wt\%=\frac{M_{1}}{M_{1}+M_{2}}\times 100$$

where *wt*% is percentage of weight. *M_1_* is mass for sample 1 and *M_2_* is mass for sample 2. The composition of niobium for reinforcement is outlined in Table 2.

**Figure 1:**
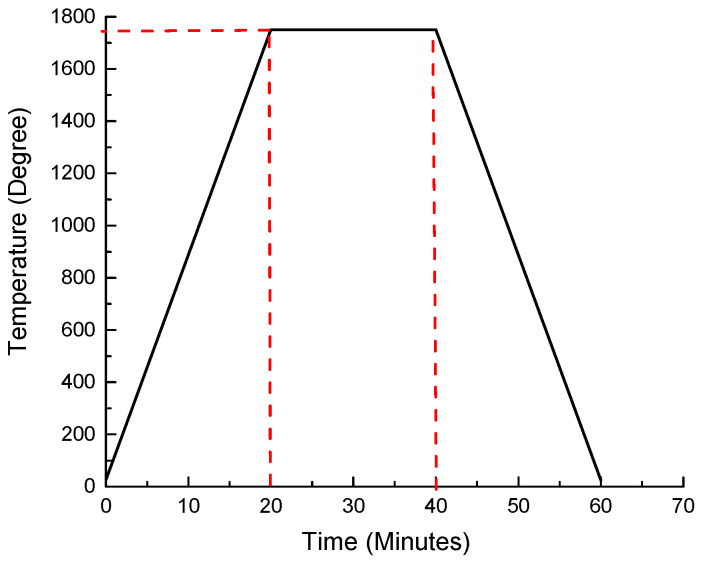
Melting profile of temperature for low-carbon steel type A333 Grade 6.

The melting process commenced utilizing an electric induction furnace. The section of low-carbon steel, specifically A333 Grade 6, was positioned within a graphite crucible on the induction coil. The furnace was set to operate at a frequency of 600 Hz, with a temperature of 1750 °C. After magnetizing the induction coil for 20 min, it initiated the heating and melting of the segment sample within the crucible. It was observed that the segment sample completely melted within this 20 min duration. Following this, the pre-measured niobium metal powder was introduced during the initial melting phase. An iron rod was utilized to stir the mixture, ensuring thorough blending of both materials for effective reinforcement. Instant solidification and subsequent remelting of the material were performed to attain a homogeneous state and maintain optimal melting conditions. Subsequently, the Fe-C-Nb samples were cast using the lost-foam casting method, taking on a rectangular shape. The experiments were replicated with varied weights of niobium metal powder at 2, 4, and 6%. The temperature melting profile for low-carbon steel type A333 Grade 6 is depicted in Figure 1 [25].

To evaluate the corrosion behavior, the Fe-C-Nb samples, featuring different amounts of Nb, were subjected to naphthenic acid. The initial masses of both Fe-C and Fe-C-Nb samples were precisely measured. Subsequently, these samples were immersed in the naphthenic acid solution and maintained at a constant temperature of 200 °C for a period of 12 h. From floating production storage and offloading (FPSO) piping specification, low carbon steel materials, which are suitable for corrosive hydrocarbon operative conditions within a temperature range of 0 °C to 240 °C [26]. Any changes in the weight of each sample during this timeframe were accurately recorded. The corrosion rate (*R_c_*) of the samples was determined using equation [27]:

(2) $$R_{c}=\frac{KW}{ATD}$$

where *K* is constant (8.76 × 10^4^), *T* is time to exposure in hours, *A* is area in cm^2^, mass lost in grams, and *D* is for density in g/cm^3^. To examine the microstructural characteristics, scanning electron microscopy (SEM) was employed at an acceleration voltage of 20,000 V, enabling a significant magnification of up to 3000×. The analysis of elements and composition in the materials was conducted using energy-dispersive X-ray analysis (EDX).

## 3. Results and Discussion

In this section, pure Fe-C and Fe-C-Nb with different Nb weight percentages are characterized. The Nb weight percentages varied at 2, 4, and 6%. Changing Nb weight percentages on the samples’ corrosion behavior is observed especially on mass loss and corrosion rate. Hence, the microstructural and chemical compositional of the samples are analyzed to discover changes of samples.

### 3.1. Physical Assessment of Fe-C and Fe-C-Nb

**Figure 2:**
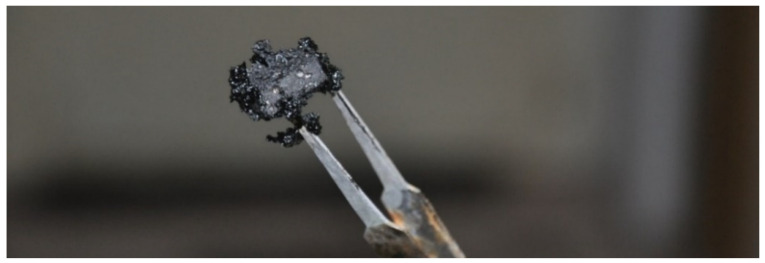
Physical assessment of Fe-C after immersion.

The Fe-C and Fe-C-Nb with different additions of Nb samples were entirely immersed in naphthenic acid sediment. Following a 12 h immersion period, all naphthenic acid transformed from a yellow hue to a black color. Additionally, it was noted that the naphthenic acid acquired a jelly-like consistency due to decomposition under the high temperature of 200 °C. Upon physical assessment, severe corrosion was evident on all samples following the rigorous corrosion experiment. The noticeable degradation on the surface was prominently identified at the sample edges, emphasizing the significant severity of naphthenic acid corrosion (NAC). The corrosion of steel in NAC is commonly linked to the creation of soluble iron naphthenates, leaving the steel surface susceptible to subsequent attacks [28]. Consequently, this condition demonstrated the corrosion impact of naphthenic acid both with and without the addition of niobium into the low-carbon steel. The physical assessment of Fe-C sample is shown in Figure 2.

### 3.2. Corrosion Analysis of Fe-C and Fe-C-Nb

This analysis encompassed the assessment of mass loss and corrosion rate after immersion. Mass loss after corrosion process denotes the decrease in the weight of a material due to the corrosive processes it experiences. This phenomenon involves the removal or dissolution of material, and its measurement involves quantifying the difference in weight of the material before and after being exposed to corrosion. Meanwhile, rate of corrosion, often referred to as the corrosion rate, is a measure of a material that undergoes corrosion over a specific period.

In the obtained results, noticeable alterations in the mass of particular samples were observed pre- and post-immersion in naphthenic acid. In the absence of any Nb addition, the pure Fe-C sample displayed a mass loss of 0.282 g. The Fe-C-Nb samples with Nb additions of 2, 4, and 6% demonstrated mass losses of 0.201, 0.324, and 0.443 g, respectively, for each corresponding sample. The investigation revealed that among the Fe-C-Nb samples, those with a 2% Nb addition exhibited the least mass loss in comparison to the others. Conversely, the Fe-C-Nb samples with a 6% Nb addition demonstrated the highest mass loss. An excess of niobium can induce alterations in the behavior of low-carbon steel in corrosive environments. The introduction of niobium may result in the formation of different precipitates or second-phase particles. If these particles do not promote corrosion resistance, their presence may contribute to an elevated level of mass loss [29].

**Figure 3:**
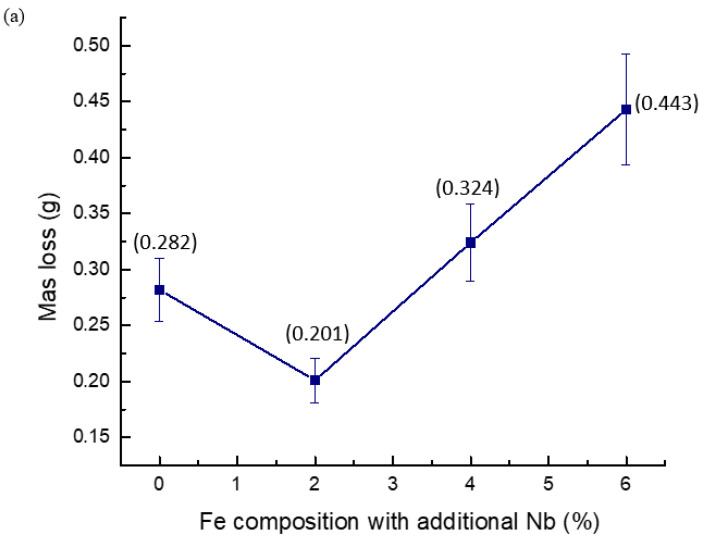

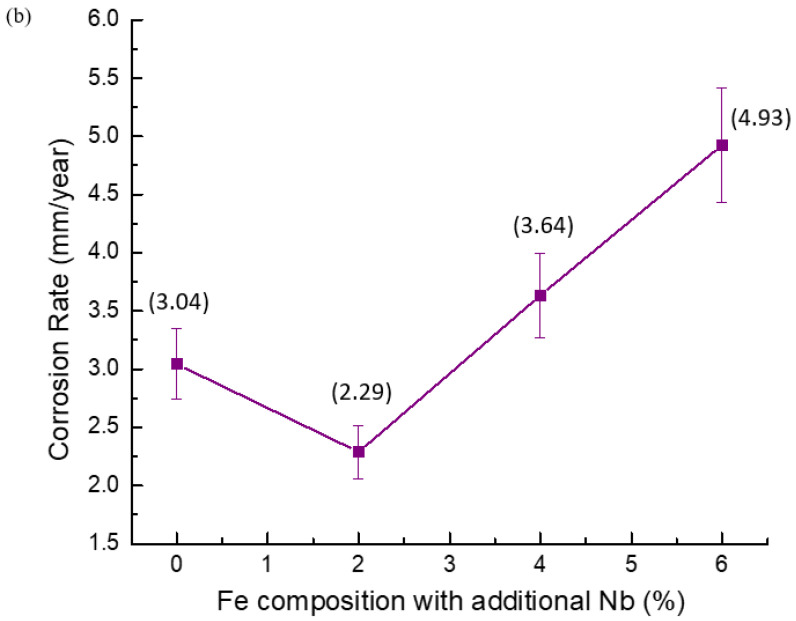
(**a**) Mass loss and (**b**) corrosion rate of Fe-C and Fe-C-Nb with different percentages of Nb addition.

The corrosion rate for the pure Fe-C sample was determined to be 3.04 mm/year. Upon introducing 2% of Nb into the sample, the corrosion rate decreased to 2.29 mm/year. However, the corrosion rates for Fe-C-Nb samples with 4% and 6% Nb additions were calculated at 3.64 and 4.93 mm/year, respectively. The results exhibited a distinct pattern, wherein corrosion rates initially decreased following the addition of 2% Nb and subsequently increased with higher Nb content. Consequently, the optimal addition of Nb was identified as 2%, as corrosion attack escalated with excessive Nb reacting with naphthenic acid. The mass loss and corrosion rate of specific sample is presented in Table 3 and Figure 3.

### 3.3. Microstructural Analysis of Fe-C and Fe-C-Nb

The microstructural examination of Fe-C and Fe-C-Nb was conducted using scanning electron microscopy (SEM). To enable a thorough analysis, secondary images at magnifications of 1000× and 3000× were utilized to offer surface views of the samples. Numerous structural features and morphologies were observed after the samples immersed into naphthenic acid, and these alterations were linked to the progressive levels of Nb addition.

For the pure Fe-C sample, the corrosion performance is exceptionally poor compared to the others. The surface displayed numerous small white particles. This condition indicated the presence of decomposed naphthenic acid covering the samples with an oil-based sediment. Oil sediment refers to solid or semi-solid residues formed by the decomposition of naphthenic acid in the presence of oil. Additionally, visibly corroded constituents were apparent on the surface. The voids observed exhibited whitish cube shapes, forming large corrosion pits in alternating layers. The corrosive action of naphthenic acid led to the deterioration of iron carbide phases, contributing to the severe growth in corrosion pits. In this stage, an intense manifestation of naphthenic acid corrosion (NAC) resulted in a severe morphological condition [30].

Following the incorporation of 2% niobium into Fe-C-Nb, an improved morphological surface was observed, while small white particles, indicative of oil sediment residue, were still present on the surface. However, only small corrosion pits were identified with dispersed whitish impurities. The occurrence of corrosion pits was significantly reduced compared to the base metal of Fe-C. This can be attributed to the contamination of the alloy by naphthenic acid, initially producing needle-like impurities on the surface, and subsequently accelerating material deterioration through electrochemical reactions. The findings were consistent with the observations made by Bo Ren et al., who indicated that the addition of Nb can stabilize the passivation film on steel and decrease pitting sensitivity. Consequently, this weakens the corrosive impact on the base steel [31].

In the case of Fe-C-Nb with a 4% niobium addition, the corroded samples were enveloped by white impurities, indicative of acid sediments. Acid sediment refers to solid residues or byproducts formed during the corrosive process involving naphthenic acid. Additionally, medium-size corrosion pits were observed beneath the dispersed acid residues. However, the corrosion pits in this sample did not exhibit a severe corrosion attack compared to the base metal Fe-C. This observation may be attributed to the presence of a thick layer of acid sediment covering the surface [32]. This observation aligns with the findings of Yunzu Xu et al., where the steel exhibited numerous scattered small pits. Furthermore, the entire steel surface exhibited roughness following the pure corrosion process [33].

**Figure 4:**
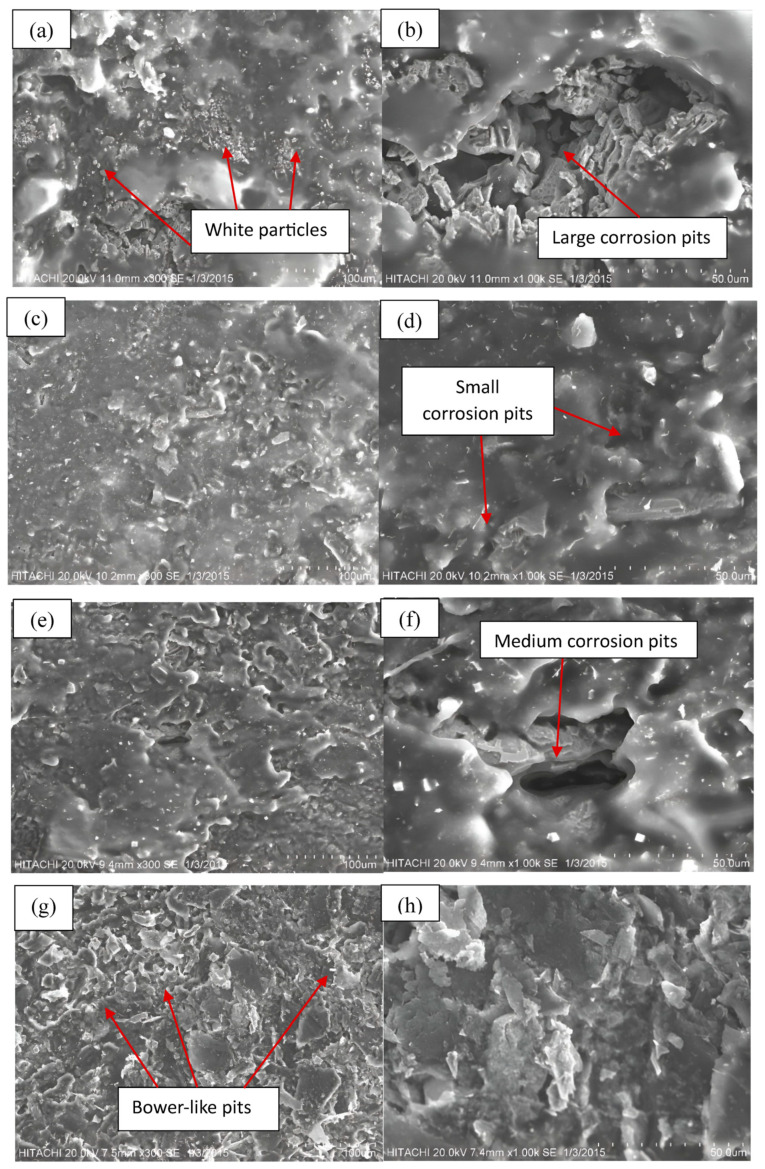
Top-view images of (**a**,**b**) Fe-C and Fe-C-Nb with (**c**,**d**) 2%, (**e**,**f**) 4%, and (**g**,**h**) 6% of Nb addition at magnifications of 1000× and 3000×, respectively.

Lastly, the surface of the Fe-C-Nb sample with a 6% niobium addition was examined. The corroded surfaces exhibited pronounced degradation owing to active corrosion. Different regions displayed a range of colors, including greyish, whitish, and dark regions, indicating diverse compositions. Furthermore, numerous corrosion pits in bower-like shapes were identified, contributing to the formation of irregular surface layers. In this case, the excessive addition of Nb into the Fe-C-Nb sample resulted in variations in the austenitizing temperature and the formation of second-phase particles. The addition of Nb was restricted to 2% due to the observation that Fe-C-Nb exhibited higher corrosion susceptibility at 4% and 6%. This finding aligns with the statement by K. Alogab et al., who suggested that beyond a certain limit of Nb percentages, maintaining a higher Nb content will not increase the amount of Nb in the dissolved state. Instead, the volume fraction of second-phase particles containing Nb will rise. With an increased volume fraction of these Nb-containing second-phase particles, the austenite transformation temperature will also increase. Consequently, the effectiveness of Nb in grain refinement becomes less pronounced with content surpassing the defined limits [34]. Top-view images of Fe-C and Fe-C-Nb with different percentages of Nb addition are shown in Figure 4.

### 3.4. Chemical Composition of Fe-C and Fe-C-Nb

By employing energy-dispersive X-ray analysis (EDX), the chemical composition of both Fe-C and Fe-C-Nb samples, with varying levels of Nb addition, was determined. This analytical process involved investigating two points on the surfaces of the samples. Through the utilization of backscattered images of the corroded samples, the relative abundance of elements at specific points in the spectrum was discerned. The elements identified included carbon, iron, oxygen, and niobium, each exhibiting different percentages.

In the case of base metal Fe-C, the first point of the spectrum was positioned in a greyish region, indicating the 72.89% of carbon. Meanwhile, the second spectrum was positioned at the whitish region resulted in 46.10% for carbon and 53.78% for iron, resembling the reduced corrosion attack that occurred. This condition is related to Equation (3), where the corrosion process occurred at high temperature.

(3) $$Fe+2RCOOH\to Fe{\left(RCOOH\right)}_{2}+H_{2}$$

where the relationship indicates that naphthenic acids oxidize iron to produce oil-soluble salts, specifically iron naphthenates, and hydrogen gas. In this reaction, ‘R’ represents the hydrocarbon segment of the naphthenic acid molecule, and ‘COOH’ is the corrosive carboxylic acid functional group [28]. This chemical reaction could potentially explain the observed increase in carbon content along with a decrease in iron for the Fe-C sample.

After addition of 2% niobium into the Fe-C-Nb sample, first spectrum was focused on a greyish region that showed a carbon content of about 71.56%. The second spectrum showed 53.28% of carbon content, indicating the lowest corrosion attack. This aligned, as the utmost level of 18.53% niobium was also detected. In addition, the morphology looks convincing as well by illustrating a whitish region similar to the morphology of Fe-C-Nb 2% before corrosion. This observation aligns with the findings of Bommareddy et al., who noted that the addition of Nb substantially increased the strength of the steel. Initially, it was believed that this enhancement was attributed to grain refinement based on solidification. Another plausible explanation was that niobium dissolved in the steel matrix and subsequently functioned as a precipitation-hardening phase [35].

In the case of Fe-C-Nb with a 4% niobium addition, the EDX analysis revealed variations in percentage composition with the increase in Nb content. In the initial spectrum, located in a whitish region, the niobium content was the highest at approximately 43.44%. Despite niobium diffusing into the ferrite phases of the material, naphthenic acid still selectively corroded iron carbides. This selective corrosion resulted in the complete absence of iron in that specific point on the spectrum, attributed to the high niobium content [36]. Furthermore, the second spectra were characterized by carbon content, measuring around 72.50%. In instances where carbon content is high, oxidation reactions may occur, leading to substantial iron loss. This observation is substantiated by the notably low iron percentages observed in this spectrum.

**Figure 5:**
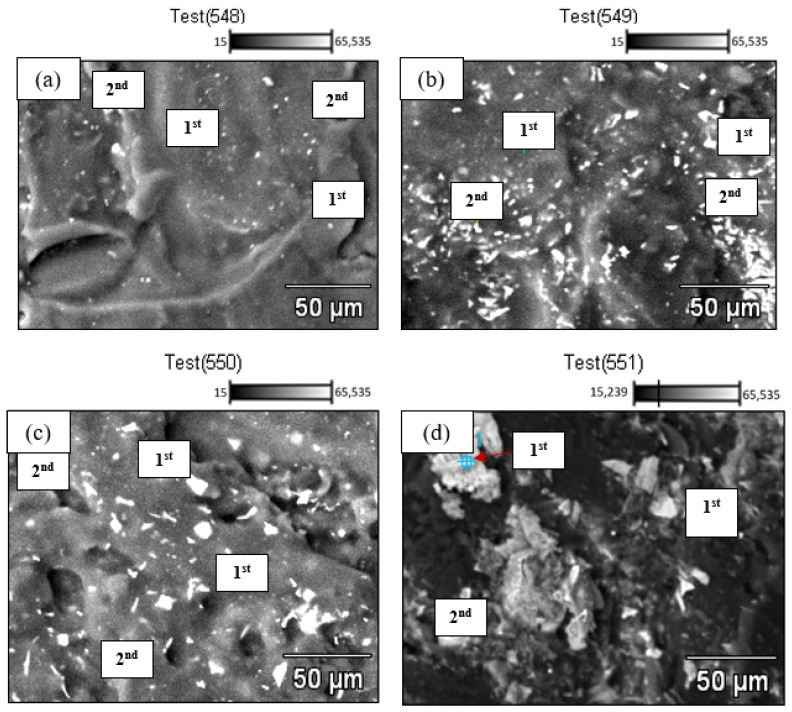
Backscattered images of (**a**) Fe-C and Fe-C-Nb with (**b**) 2%, (**c**) 4%, and (**d**) 6% percentages of Nb addition.

In the investigation of Fe-C-Nb with a 6% niobium addition, three spectra were obtained from several regions to discern the impact of naphthenic acid. In the first spectrum, found within the greyish region, carbon content measured at 28.76%, while iron content stood at 70.93%, accompanied by a minimal niobium content of only 0.32%. In contrast, the second point in the whitish phase comprised 14.05% of carbon and constituted 85.95% of niobium from the spectrum. Both spectra indicated evidence of corrosion attack. However, the absence of iron elements in the whitish phase was attributed to the excessive presence of niobium elements. The findings are consistent with a prior study conducted by Zong et al., which indicated that the increase in Nb addition corresponds to a concurrent decrease in carbon. Lower carbon levels contribute to improved tightness and weldability. However, it is crucial to maintain a moderate range of niobium content, as excessive amounts can have implications on the material’s properties [37]. Backscattered images and chemical composition of Fe-C and Fe-C-Nb with different percentages of Nb addition are presented in Figure 5 and Table 4.

## 4. Conclusions

The findings reveal that subjecting Fe-C, specifically A333 Grade 6, to naphthenic acid solution leads to material impacts. Among the various percentages of Nb additions, it was conclusively demonstrated that adding 2% niobium to Fe-C is the most effective in reducing the corrosion rate of the material. Fe-C-Nb: 2% addition showed the least mass loss and in corrosion rate detected after a 12 h immersion in naphthenic acid. In terms of microstructure, after incorporating 2% niobium, the occurrence of corrosion pits notably diminished in comparison to the Fe-C sample, with only small corrosion pits being observed. However, with an increase up to 6% niobium, numerous voids in bower-like shapes were identified, suggesting that the addition of niobium should be limited to only 2%. Furthermore, the chemical composition analysis of the Fe-C-Nb: 2% addition spectrum revealed a carbon content of 53.28%, indicating a low level of corrosion attack. This suggests that the niobium dissolved in the steel matrix and subsequently functioned as a precipitation-hardening phase. Thus, the superior resistance to severe acidic environments and conditions was primarily limited to Fe-C-Nb 2%. Therefore, Fe-C-Nb with a 2% Nb addition can be considered superior in corrosion resistance to low-carbon steel type A333 Grade 6 for offshore platform pipeline applications.

## Figures and Tables

**Table 1:** Chemical composition and mechanical characteristics for low-carbon steel type ASTM A333 Grade 6.

Chemical composition	Carbon (C)	:	0.30% max
	Manganese (Mn)	:	0.29–1.06%
	Phosphorus (P)	:	0.025% max
	Sulfur (S)	:	0.025% max
	Silicon (Si)	:	0.10% min
	Nickel (Ni)	:	3.18–3.82%
	Chromium (Cr)	:	0.40% max
	Copper (Cu)	:	0.40% max
	Vanadium (V)	:	0.08% max
Mechanical characteristics	Tensile Strength	:	415 MPa (60,200 psi) minimum
	Yield Strength:	:	240 MPa (35,000 psi) minimum
	Elongation:	:	30% minimum (in 2 inches)
	Hardness:	:	Not specified, typically falls within the range of 137–207 Brinell or 74–148 Rockwell B hardness

**Table 2:** Composition of niobium for reinforcement.

Weight of Percentage (%)	Mass (g)
2	2.24
4	4.48
6	6.72

**Table 3:** Mass loss and corrosion rate of Fe-C and Fe-C-Nb with different percentages of Nb addition.

Composition	Initial Mass (g)	Final Mass (g)	Mass Loss (g)	Corrosion Rate (mm/Year)
Fe-C	3.871	3.589	−0.282	3.04
Fe-C-Nb: 2% addition	2.502	2.301	−0.201	2.29
Fe-C-Nb: 4% addition	4.443	4.119	−0.324	3.64
Fe-C-Nb: 6% addition	4.923	4.480	−0.443	4.93

**Table 4:** Chemical composition of Fe-C and Fe-C-Nb with different percentages of Nb addition.

Composition	Elements				
	Points	Carbon, C (%)	Iron, Fe (%)	Oxygen, O (%)	Niobium, Nb (%)
Fe-C	1st	72.89	9.13	17.78	-
	2nd	46.10	53.78	-	-
Fe-C-Nb: 2% addition	1st	71.56	-	20.30	8.14
	2nd	53.28	12.46	15.72	18.53
Fe-C-Nb: 4% addition	1st	43.44	-	11.75	44.81
	2nd	72.50	-	16.25	44.81
Fe-C-Nb: 6% addition	1st	28.76	70.93	-	0.32
	2nd	14.05	-	-	85.95

## Data Availability

The raw data supporting the conclusions of this article will be made available by the authors on request.
